# Advancements in Stem Cell Applications for Livestock Research: A Review

**DOI:** 10.3390/vetsci12050397

**Published:** 2025-04-23

**Authors:** Limeng Shi, Muhammad Zahoor Khan, Abd Ullah, Huili Liang, Mingyang Geng, Muhammad Faheem Akhtar, Jincheng Na, Ying Han, Changfa Wang

**Affiliations:** 1 Liaocheng Research Institute of Donkey High-Efficiency Breeding and Ecological Feeding, College of Agriculture and Biology, Liaocheng University, Liaocheng 252000, Chinazahoorkhan@lcu.edu.cn (M.Z.K.);; 2 Yili Kazak Autonomous Prefecture Livestock General Station, Xinjiang Autonomous Region, Yili 835000, China

**Keywords:** animal welfare, herbivores, mastitis, orthopedic diseases, regeneration, stem cells

## Abstract

Stem cells (SCs), distinguished by their capacity for self-renewal and multipotent differentiation, represent a cornerstone of regenerative medicine. These cells, which can be categorized according to their differentiation potential and developmental origin, have emerged as pivotal elements in both biomedical research and veterinary science. In herbivorous species, stem cell applications have yielded particularly promising advances across multiple domains, including reproductive biotechnology, tissue engineering and regeneration, therapeutic interventions, and immunomodulation. This review synthesizes contemporary research on stem cell biology in five economically significant herbivorous species: bovine, ovine, deer, equine, and camelid. Special emphasis is placed on stem cell isolation methodologies, culture optimization techniques, and the molecular mechanisms governing key signaling pathways. The discussion encompasses both the technical impediments facing stem cell research and the ethical framework necessary for responsible scientific advancement, with particular attention to animal welfare considerations in the development and implementation of stem cell-based technologies.

## Simple Summary

This review examines the current status and future prospects of stem cell applications in livestock research, focusing on herbivorous species, including bovines, goats, deer, horses, and camels. This review explores various types of stem cells (induced pluripotent, embryonic, and mesenchymal) and their therapeutic applications in treating conditions, like mastitis in cattle, muscle development in goats, tissue regeneration using deer antler stem cells, and orthopedic issues in horses. The authors discuss isolation methodologies, culture techniques, and the molecular mechanisms governing stem cell function. This review also addresses challenges in the field, including technical difficulties, genetic stability concerns, and ethical considerations related to animal welfare in stem cell research.

## 1. Introduction

Stem cell research has emerged as a pivotal and rapidly evolving field within the life sciences, garnering significant attention for its transformative potential in biomedical applications [1,2]. Stem cells possess the unique abilities of self-renewal and pluripotency, enabling them to differentiate into a diverse range of specialized cells under appropriate conditions [3]. These remarkable properties hold immense promise for advancing therapeutic strategies in tissue regeneration, disease treatment, and the development of biopharmaceuticals [4,5,6]. In parallel, herbivores, an essential component of global animal husbandry, play a crucial role in sustaining human life and fostering social and economic development. The application of stem cell technologies to herbivore research has proven to be a significant area of study, with the potential to enhance productivity, improve animal welfare, and promote the sustainable development of animal husbandry practices [7,8,9,10,11,12].

Over recent decades, advancements in molecular biology, cell biology, and genetic engineering have led to notable breakthroughs in stem cell science. These innovations have facilitated the identification and characterization of various stem cell types, including totipotent, pluripotent, multipotent, oligopotent, and unipotent stem cells, each with distinct differentiation capacities and replication potentials [13]. In the study of herbivore stem cells, researchers have developed sophisticated methods for isolation and cultivation. Through the utilization of specific media and growth factors, the proliferation and differentiation of herbivore stem cells can be effectively induced [14,15]. Additionally, gene editing technologies have been instrumental in stem cell research, particularly in elucidating the roles of specific genes in stem cell self-renewal and differentiation processes [16]. These discoveries not only offer new avenues for clinical applications but also provide essential tools for understanding the fundamental processes of life itself.

The applications of stem cells in herbivore research have demonstrated remarkable progress across multiple domains. Stem cell technology shows considerable promise in enhancing reproductive efficiency, facilitating the inheritance of superior genetic traits, and improving overall production performance [17,18]. Furthermore, stem cell therapies have shown significant potential in addressing common breeding-related injuries affecting bone, muscle, and skin tissues through targeted differentiation into specific cell types [7,19,20,21,22]. The immunomodulatory properties of stem cells have also proven valuable in regulating immune responses, reducing inflammation, and treating various immune and infectious diseases in herbivores [23,24,25,26]. Notably, herbivore stem cell research has opened new possibilities in human regenerative medicine and organ transplantation, as exemplified by studies on liver regeneration [27].

This review aims to: (i) systematically analyze and consolidate current research advancements in stem cell applications across herbivore species; (ii) critically evaluate the therapeutic potential of stem cells in enhancing animal welfare, production performance, and disease management; and (iii) provide valuable insights to guide future scientific investigations and facilitate the practical integration of stem cell technologies in both research and industrial applications.

## 2. Overview of Stem Cells and Their Biological Function

**Figure 1:**
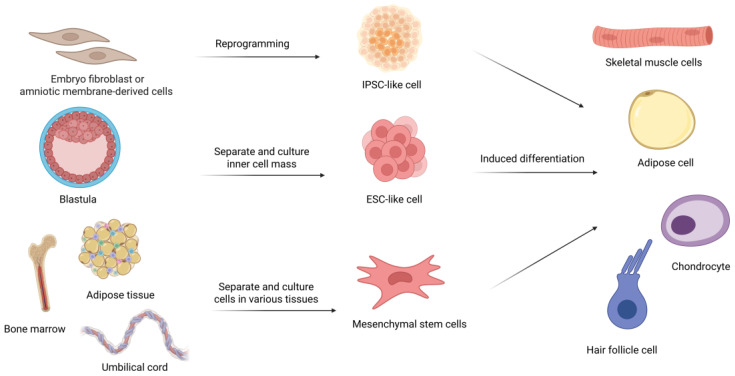
Sources and differentiation pathways of major stem cell types. The figure illustrates three main categories of stem cells and their differentiation potential. Embryo fibroblast or amniotic membrane-derived cells undergo reprogramming to become Induced pluripotent stem cells (iPSC-like cells), which can then differentiate into skeletal muscle cells, adipose cells, hair follicle cells, and chondrocytes. A blastula is used to separate and culture inner cell mass, resulting in Embryonic stem cells (ESC-like cells). These undergo induced differentiation to form the same specialized cell types shown on the right. Furthermore, tissues such as bone marrow, adipose tissue, and umbilical cord can have their cells separated and cultured to produce mesenchymal stem cells (MSCs). This figure effectively captures the main sources of stem cells (embryonic, induced pluripotent, and adult mesenchymal) and demonstrates how these different starting materials can be processed to create various specialized cell types for research or therapeutic applications.

Stem cells are unspecialized cells with the unique ability to self-renew and differentiate into specialized cell types under the right conditions. Their potential to generate diverse cell types makes them a key focus in regenerative medicine, tissue engineering, and developmental biology. Based on their origin, stem cells can be classified into three main types: induced pluripotent stem cells (iPSCs), embryonic stem cells (ESCs), and mesenchymal stem cells (MSCs) (Figure 1).

The iPSCs, which are generated through reprogramming somatic cells with specific pluripotency factors, have emerged as crucial tools for investigating animal diseases, and these cells are fundamental in regenerative medicine, disease modeling, drug discovery, cell therapy, and gene therapy [25,28,29]. Consistently, ESCs have been successfully established and obtained from various herbivores such as bovine and goat. Currently, researchers are continually striving to optimize culture conditions for these ESCs, which hold significant promise for implementation in future medical interventions [30,31,32,33]. While MSCs are multipotent stem cells characterized by their self-renewal capacity and ability to differentiate into various tissue types, these cells can be isolated from multiple sources, including bone marrow, adipose tissue, umbilical cord, and placental tissues [11,34,35,36]. MSCs demonstrate the capability to differentiate into osteoblasts, chondrocytes, and adipocytes, among other cell types. Their secretome, comprising cytokines, chemokines, and growth factors, plays a crucial role in regenerative and immunomodulatory functions [37]. Bone marrow-derived MSCs (BM-MSCs) have demonstrated particular efficacy in clinical trials for OA treatment, showing potential in cartilage repair and regeneration through their multilineage differentiation potential and immunosuppressive capabilities [1,38,39]. Furthermore, MSCs exhibit significant anti-aging applications through their anti-inflammatory, antioxidative, proangiogenic, antitumor, antifibrotic, and antimicrobial effects, which contribute to combating aging-related diseases [40,41].

Stem cells are categorized based on their differentiation potential into five distinct types: totipotent, pluripotent, multipotent, oligopotent, and unipotent [13]. These classifications are essential for understanding the developmental potential of stem cells and their roles in both normal physiological processes and regenerative medicine. Totipotent stem cells represent the most versatile category, capable of differentiating into all cell types, including both embryonic and extra-embryonic tissues, giving rise to a complete organism. In mammals, this totipotent state is exclusively observed in the zygote and the two-cell stage embryo’s blastomere, as these are the only cells capable of generating both the embryo and its supporting tissues such as the placenta [42]. On the other hand, pluripotent stem cells can differentiate into cells from the three primary germ layers, endoderm, mesoderm, and ectoderm, allowing them to form a wide variety of specialized cell types, and embryonic stem cells are a key example of pluripotent cells. Their differentiation capabilities make them highly valuable for applications in regenerative medicine and disease research [43].

Multipotent stem cells can differentiate into a limited range of cell types, usually confined to a specific lineage. The MSCs serve as a prime example, as they can develop into various cell types, such as osteoblasts, chondrocytes, adipocytes, and tendon cells. This capacity to generate multiple cell types within a specific lineage makes MSCs particularly useful in tissue repair and regenerative therapies [44,45]. Oligopotent stem cells have a more restricted differentiation potential compared to multipotent cells, as they can only give rise to a limited range of cell types within a specific tissue. For instance, the common myeloid progenitor (CMP) can differentiate into granulocyte–monocyte progenitors (GMPs), which then form granulocytes and monocytes, and into megakaryocyte-erythroid progenitors (MEPs), which produce platelets and red blood cells [46]. Unipotent stem cells are the most specialized, only able to differentiate into one specific cell type. Spermatogonial stem cells exemplify this category, as they only produce male gametes. Despite their limited differentiation capacity, unipotent stem cells play essential roles in maintaining tissue homeostasis and supporting specific physiological functions [47].

Stem cells, owing to their unique biological properties, have become a key cornerstone in regenerative medicine, offering promising solutions for various intractable diseases. Their therapeutic potential derives from their ability to differentiate into specific cell types through bioengineering techniques, aiding in the repair of damaged tissues. Notably, stem cells have shown significant promise in regenerating orthopedic tissues such as cartilage (chondrogenesis), tendons, and bones. This makes them a viable treatment option for conditions like osteoarthritis and mandible defects [10,19,29,48,49,50,51,52]. Additionally, stem cell-based therapies have been pivotal in nerve regeneration, particularly with neural crest cells (NCC), which have demonstrated potential for repair of the nervous system [53]. Furthermore, stem cells are increasingly recognized for their therapeutic roles in metabolic diseases, such as diabetes, where they offer potential for regenerative treatments [54,55].

## 3. Research Development on Herbivore Stem Cells

In recent years, the application of stem cell technology in herbivores has gradually become a research hotspot. For example, MSCs and ESCs can be used to repair damaged tissues, such as tendon tissue and mammary tissues, thus improving the health level of animals and prolonging the production life of animals [1,56,57,58]. Goat stem cells, including embryonic and induced pluripotent stem cells, are being studied to boost milk, meat, and fiber production. They also serve as models for researching human diseases [59]. In addition, scientists are still working to optimize the reprogramming process [60] and stem cell culture conditions [61] and, in doing so, expect to produce herbivores with superior traits, and MSCs derived from herbivores such as forest musk deer and goats are vital for regenerative therapies, tissue repair, germplasm preservation of endangered species, disease modeling, and advancements in regenerative medicine [29,52,62,63,64]. Stem cells are increasingly used in veterinary medicine for treating musculoskeletal injuries in horses [65]. Bovine stem cells, including embryonic stem cells (bESCs) and induced pluripotent stem cells (biPSCs), enhance breeding and reproduction by enabling in vitro differentiation modeling, disease and cancer studies, and supporting genetic engineering and cloning [66,67]. The applications of various stem cells in herbivorous livestock research are summarized in Table 1.

### 3.1. Bovine Stem Cell Research

Bovine embryonic stem cells (bESCs) are a kind of cell with self-renewal ability and the ability to differentiate into all three kinds of germ layer cells. They mainly come from trophoblast cells of early embryos and show a high level of self-renewal ability under the condition of in vitro culture, and they can be passed down through cell culture without losing their pluripotency [68,69]. Although the scheme of inducing and culturing long-term stable embryonic stem cells in cattle is still being optimized, bovine embryonic stem cells are the first ESC for livestock and poultry that can be obtained in vitro and maintain a pluripotent state. The culture medium of the custom TeSR1 base medium (growth factor-free) supplemented with FGF2 and IWR1 (CTFR) used by Bogliotti et al. can maintain the pluripotency and long-term proliferation of pluripotent bESCs, and IWR1 is a classical WNT signal transduction pathway inhibitor. It is proved by experiments that its addition is very important for the derivation of bESCs [32]. Since then, researchers have continuously optimized the composition of culture media and the source of cells for more efficient and stable passage. For example, when using the N2B27 basal medium supplemented with BSA instead of the mTeSR1 basal medium, bESCs can still be effectively derived, and self-renewal and pluripotency can be maintained [70]. At the same time, the method of reprogramming bovine somatic cells into induced pluripotent stem cells (iPSCs) and expanded potential stem cells (EPSCs) is also developing. Induced pluripotent stem cells (iPSCs) are pluripotent cells produced by reprogramming differentiated cells such as fibroblasts. Researchers transduced fibroblasts by constructing virus vectors carrying specific genes and induced them to be reprogrammed into iPSCs. IPSCs have similar pluripotency to embryonic stem cells and can differentiate into various cell types. In 2011, for the first time, researchers successfully generated biPSC cell lines from bovine embryonic fibroblasts by transducing six transcription factors, namely OCT4 (also known as POU5F1), SOX2, KLF4, MYC, LIN28 and NANOG [71]. The method of reprogramming bovine somatic cells into biPSCs is developing continuously. Zeng et al. successfully constructed a lentiviral vector (LV) carrying the pluripotent gene in yak for the first time. The vector effectively delivered the gene to yak fibroblasts, and it was verified that the treated cells had various pluripotent characteristics. This study laid a foundation for establishing yak iPSC lines induced by specific yak transcription factors and further exploring yak iPSCs, which was of great significance for yak variety improvement and germplasm resource protection [60]. Mesenchymal stem cells, as an adult stem cell with self-renewal ability and multidirectional differentiation potential, can be extracted from bovine bone marrow, fat, periosteum and other tissues, and can be differentiated into bone, cartilage, fat and muscle and other cell types. MSCs can be used to treat tissue injuries, such as bone, cartilage, fat and muscle, as well as some hereditary diseases, degenerative diseases and infectious diseases.

#### 3.1.1. Role of Stem Cells in Mastitis Treatment in Bovine

Mastitis, an inflammation of the mammary glands, leads to substantial economic losses worldwide, such as reduced milk production, increased veterinary costs, and decreased milk quality in dairy cattle [24]. Mesenchymal stem cells (MSCs), due to their self-renewal, differentiation capabilities, and ability to migrate to injury sites, hold potential as a therapeutic approach for repairing damaged mammary tissue [69,70,72,73]. The antibacterial properties of MSCs isolated from fetal bovine bone marrow (BM-MSCs) and adipose tissue (AT-MSCs) against Staphylococcus aureus have been demonstrated in vitro using conditioned medium (CM) [35]. Experimental findings reveal that the mRNA expression and protein levels of the antibacterial peptide gene bBD4A are upregulated in MSCs from both tissues following direct exposure to S. aureus. However, the upregulation of NK1 mRNA expression is observed exclusively in AT-MSCs, indicating that the antibacterial mechanisms of MSCs are tissue-specific [35]. Furthermore, the immune response to bacterial infections also exhibits species specificity. Indoleamine 2,3-dioxygenase (IDO), a type of antimicrobial peptide, has been shown to be active in human MSCs when stimulated by proinflammatory cytokines and IFN-γ. In contrast, under similar conditions, murine MSCs do not exhibit the same IDO-mediated antibacterial effect [74]. A study assessing the efficacy of intramammary MSC therapy for bovine mastitis demonstrated that repeated intramammary administration of allogenic bovine fetal AT-MSCs did not induce immune rejection or memory in healthy dairy heifers. Additionally, the treatment led to a reduction in bacterial counts in milk from cows with S. aureus-induced mastitis compared to those treated with a vehicle control [75]. These findings support the potential of stem cells as a therapeutic option for mastitis. However, challenges remain, including the risk of immune rejection associated with allogeneic stem cell transplantation and variations in the degree of mastitis and recovery potential among individual bovines.

#### 3.1.2. Role of Stem Cells in Cultured Meat in Bovine

Stem cells play a crucial role in the production of cultured meat, a process that involves cell engineering in vitro and offers significant benefits in terms of environmental sustainability, food safety, and animal welfare [76]. Currently, the primary sources of stem cells for cultured meat production are muscle tissue [77,78], adipose tissue [79,80] and umbilical cord tissue [81]. These stem cells, selected for their high proliferative capacity, can be directed to differentiate into specific tissue types on plant-derived scaffolds that mimic the properties of natural tissue [82]. The resulting tissue blocks are then assembled in specific proportions to form cultured meat.

In efforts to enhance the safety, taste, flavor, nutritional quality, and cost-effectiveness of cultured meat, researchers are continuously optimizing stem cell sources, differentiation media, and scaffold compositions. Additionally, gene editing is being used to regulate nutritional components. For example, bovine umbilical cord stem cells have been used for adipogenic differentiation, with non-invasive methods employed to reduce the harm caused by biopsies on animals [81]. A study demonstrated that fetal bovine serum (FBS) substitutes derived from the blood of mature livestock slaughterhouses can significantly reduce the production costs of cell culture media, contributing to the large-scale production of cultured meat [83]. Furthermore, the use of *Grifola frondosa* extract as an ingestible additive has been shown to promote cell proliferation while decreasing the amount of FBS required during the culture process [84]. Given the critical role of 3D scaffolds in cultured meat production, researchers have also explored the ethical issues related to their sourcing, as well as their safety and taste as food. For instance, gelatin and placenta tissue have been studied as potential host scaffolds [85,86]. However, despite these advancements, concerns regarding the potential health risks of cultivated meat and consumer acceptance remain, necessitating further investigation and consideration [87].

### 3.2. Goat Stem Cell Research

There are three main sources of goat stem cells, namely embryonic stem cells, adult stem cells and induced pluripotent stem cells. Behboodi et al. isolated goat embryonic stem cell lines from goat embryos for the first time, which can detect the expression of known ES cell markers. The isolated ES cells can differentiate into ectodermal cells in vitro and form teratomas after being transplanted into immunocompromised mice [30]. Wei et al. isolated inner cell mass (ICM) in vitro through micromanipulation and inoculated the isolated inner cell mass into a 2i medium, 2i+LIF medium and control medium, respectively. After culture, it was found that the goat ICM-derived cell subculture in the 2i+LIF medium had mouse ES-like morphology, but its proliferation ability was limited. However, in a 2i single culture medium, the goat ICM-derived cells had primate ES-like morphology in the first generation, but most of them had obvious differentiation in the first passage, and these cells expressed pluripotent markers. However, after a period of time, the pluripotency of goat inner cell mass cells in the suspension culture medium decreased gradually, and the markers of three germ layers could be detected. Moreover, goat ICM-derived cells may be derived from ectoderm [88]. Based on the proliferation ability and multi-lineage differentiation ability of embryonic stem cells, they can be directionally differentiated into germ cells, thus improving the symptoms of infertility [89]. Goat spermatogonial stem cells (SSCs) are adult pluripotent stem cells, so it is very important to choose a more efficient way to obtain them and a storage method that can maintain their survival status and functional characteristics for a long time. Pramod et al. used three enzymes (collagenase IV, trypsin and DNase I) to digest and separate spermatogonial stem cells in goat testis for the first time and then used Percoll density gradient centrifugation technology to further enrich them [90]. Saleema Ahmedi Quadri and others identified the total cell number and cell survival rate after thawing and proved that the cell survival rate of spermatogonial stem cells in the culture system was higher when trehalose was used for cryopreservation, and the cell proliferation potential after thawing was higher [91]. At the same time, genes related to the renewal and proliferation of spermatogonial stem cells have also attracted the attention of scientists, such as Nanos2 [92] and Lin28a [93,94]. And, since the first report of sheep iPSCs was published in 2011 [95,96], scientists have put a lot of effort into inducing sheep IPSCs, including cell types that can be used for reprogramming, using different culture conditions and different transgenic methods.

#### 3.2.1. Regulation Mechanism Related to Muscle Development Based on Non-Coding RNAs

The development of skeletal muscle directly influences both the quantity and quality of the meat produced [97]. The dynamic development and repair of skeletal muscle rely on the self-renewal and differentiation of muscle satellite cells (MuSCs), a type of muscle stem cell located beneath the basement membrane of muscle fibers [98,99]. The regulation of non-coding RNA, particularly microRNAs (miRNAs), plays a pivotal role in the biological processes of MuSCs [100]. Recent studies have provided significant insights into the role of miRNAs in skeletal muscle development. For instance, miR-193b-3p has been found to be highly expressed in goat skeletal muscle, with its deficiency impairing the proliferation and differentiation of myoblasts. Conversely, the ectopic expression of miR-193b-3p promotes both the proliferation and differentiation of these cells. This study also highlighted the miR-193b-3p/IGF2BP1 axis as a key regulatory mechanism for myoblast proliferation [101].

In addition, miR-381 has been shown to promote the myogenic differentiation of MuSCs by up-regulating the expression of myogenic differentiation marker genes such as MyHC, MyoG, and MEF2C. The reduced expression of miR-381 disrupts the Notch signaling pathway, which, in turn, inhibits the proper proliferation and differentiation of MuSCs [102]. Circular RNAs (circRNAs), a class of single-stranded non-coding RNAs, also play important roles in regulating MuSC function. For example, circTGFβ2 was identified through RNA sequencing and gene function enrichment analysis as a circRNA that promotes the myogenic differentiation of myoblasts [103]. Furthermore, circ_14820, enriched in the longissimus dorsi muscle, significantly promotes MuSC proliferation but inhibits differentiation when overexpressed [104]. Other mechanisms, such as the regulation of myogenic regulators, RNA-binding proteins, and RNA editing sites, are also critical areas of investigation for understanding muscle development at the molecular level [105,106,107].

#### 3.2.2. Regulation of Hair Follicle Development via Multiple Signaling Pathways

Cashmere, a highly valuable textile material, is primarily produced through the growth of the secondary hair follicle (SHF) in goats [17]. The biological cycle governing SHF development is critical to the formation and growth of cashmere fibers. CircRNAs have been shown to regulate the differentiation of hair follicle stem cells (HFSCs), influencing SHF regeneration and the formation of cashmere fibers [108]. For example, circERCC6 positively regulates the activation of secondary hair follicle stem cells (gsHFSCs) in cashmere goats, with m6A modification of circERCC6 being essential for its action through the miR-412-3p/BNC2 pathway [109]. Similarly, circRNA-1926 promotes the differentiation of SHF stem cells into hair follicle lineages by regulating the miR-148a/b-3p/CDK19 axis [108]. Both BNC2 and CDK19 are involved in the Wnt signaling pathway, which is crucial for stem cell regulation and plays a significant role in hair follicle development. Additionally, the Wnt signaling pathway has been shown to influence melatonin-mediated stemness in gsHFSCs by regulating NOGGIN [8,18] These findings highlight the complex interplay between circRNAs, miRNAs, and signaling pathways in regulating both muscle and hair follicle development, which are essential for improving livestock traits such as meat quality and fiber production.

### 3.3. Deer Stem Cell Research

Deer antlers represent a unique example of mammalian structural regeneration, with the ability to regenerate throughout the life cycle of the animal. They can be completely regenerated from the pedicle after being lost. This regenerative capability is attributed to the self-renewal and differentiation potential of antler stem cells (ASCs) [110]. There are three primary sources of these stem cells: antlerogenic periosteum (AP), pedicle periosteum (PP), and reserve mesenchyme (RM). AP cells proliferate and differentiate under the influence of androgen hormones and growth factors to form pedicles and initial antlers [111]. Subsequently, the periodic renewal of antlers is initiated by the activation of PP cells [112]. These PP cells, which possess high proliferative capacity, support rapid antler growth and can differentiate into chondrogenic lineages [113]. The regeneration of antlers is regulated by several factors, including cell cycle regulation, growth factors and cytokines, and cellular signaling pathways. Antler genesis and antler regeneration are regulated by androgen, which changes according to the cyclic changes of androgen in deer [114]. In addition, it has been found that galectin-1 (GAL-1) is a kind of protein that is highly expressed in regenerative tissues such as the periosteum of raw antlers, pedicled periosteum and the growth center of antlers’ tip, and in antlers’ stem cells. Therefore, Li et al. conducted in-depth research on the role of galectin-1 in antlers’ regeneration and verified through experiments that deer GAL-1 protein is highly expressed in ASCs, which can regulate its differentiation and has strong angiogenesis activity [115]. Guo et al. discovered the tumor suppressor gene BRCA1 when they explored the candidate factors that can maintain the rapid proliferation and genome stability of the activated antler stem cells (AcAnSCs). In this study, researchers verified its function by knocking out the BRCA1 gene. It was verified that the high expression of BRCA1 in the activated antler stem cells may be related to maintaining the rapid proliferation and genome stability in the process of antler regeneration and can effectively improve the cell viability [116].

The regenerative process of antlers, which includes the repair of bone, skin, blood vessels, and nerves, is a model of wound healing and stem cell-mediated regeneration [117,118,119]. Therefore, current research on deer stem cells focuses on promoting wound healing, reducing scarring, and enhancing bone formation [7,120,121]. Fibroblasts are key players in wound healing; however, the fibroblast-to-myofibroblast transition (FMT) is crucial for scar formation [120]. An antler stem cell-conditioned medium (ASC-CM) has been identified as a promising therapeutic agent. Studies show that ASC-CM stimulates the proliferation of human umbilical vein endothelial cells (HUVECs) and NIH-3T3 cells, promoting wound healing. The expression ratios of key regulatory genes, such as Col3A1/Col1A2, TGF-β3/TGF-β1, MMP1/TIMP1, and MMP3/TIMP1, were found to be similar to those in fetal healing tissues [7]. Furthermore, antler stem cell-derived exosomes (AnSC-exos) inhibit FMT by down-regulating TGF-β1 signaling, improving wound healing quality [120]. Exosomes derived from antler mesenchymal stem cells (AMSC-Exo) enhance specific miRNAs related to cell proliferation and angiogenesis, modulating signaling pathways such as MAPK, PI3K-Akt, and HIF-1, thereby accelerating wound healing and reducing scar formation [121].

In addition to wound healing, deer stem cells have been explored for their potential in treating osteochondral defects and periodontal tissue repair. Periodontitis, an infection of periodontal tissues that can lead to tooth loss, originates from cranial nerve crest cells (CNCCs) [53,122]. Interestingly, antler stem cells (AnSCs), which also derive from neural crest cells, have shown superior efficacy compared to bone marrow-derived mesenchymal stem cells (BMSCs) and adipose-derived MSCs (AMSCs) in repairing periodontal tissue damage [21,123]

### 3.4. Horse Stem Cell Research

With the progress of gene editing technology such as CRISPR/Cas9, researchers have carried out lots of basic research based on the editability of embryonic stem cells. OCT4 is a transcription factor that is highly expressed in early embryos, and, based on its indispensability in embryonic stem cell formation, it is also a pluripotent marker of stem cells in horses and other species. Erin Hisey and others found that OCT4 protein and POU5F1 gene encoding OCT4 protein are highly conserved among species through NCBI protein BLAST (ElasticBLAST 1.4.0, 17 March 2020) comparison, but after research, it is difficult to establish an in vitro model of conditional knock-out based on Oct4, so it is also worthy of attention to further study the function of OCT4 protein in horse stem cells and isolate embryonic stem cells with greater pluripotent potential [124]. Mesenchymal cells also have great potential in the therapeutic application of horses, such as the treatment of endometrial inflammation [125,126,127] and corneal ulceration [128]. At present, the main sources of mesenchymal stem cells are bone marrow and fat, but researchers are looking for a more suitable sampling method based on the possibility that invasive sampling may introduce postoperative infections to animal puncture sites and the consideration of animal welfare. For example, Mohamad Khir Shikh Alsook et al. isolated and expanded the living mesenchymal stem cell population from the suspensory ligament (SL) of a horse within 72 h after death and verified the dryness of the cells obtained by them through experiments, which showed that obtaining a specific stem cell population from the ligament of a horse within 72 h after death is a source of stem cells that can be used for regenerative medicine and cell therapy [129]. In addition, Li et al. put forward a new method of minimally invasive collection, separation and culture of autologous horse stem cells, namely equine mesenchymal stem cells from the hair follicle outer root sheath (eMSCORS), and compared with horse adipose-derived mesenchymal stem cells (eADMSC), more cells can be amplified; this dryness has also been verified by experiments [130]. Finally, induced pluripotent stem cells also have certain applications in the treatment of tendon injury in horses. However, compared with embryonic stem cells, the induced pluripotent stem cells seem to have lower tendon differentiation potential [131].

#### 3.4.1. Application of Stem Cell Derivatives in Osteoarthritis of Horses

Osteoarthritis (OA), also known as degenerative joint disease (DJD), is one of the most prevalent causes of lameness in horses. OA is characterized by the degeneration of articular cartilage, accompanied by changes in the bones and soft tissues of the joint. In advanced stages, OA leads to the complete loss of articular cartilage, causing pain, deformity, and loss of mobility, which severely affects the health and athletic performance of horses, resulting in significant economic losses to the equine industry [132]. Inflammation plays a key role in the pathophysiology of OA [51]. Given the immunomodulatory properties of mesenchymal stem cells (MSCs) and the factors they secrete, current research is focused on using extracellular vesicles (MSC-EVs) derived from MSCs to explore their therapeutic potential for arthritis [50].

Integrin α10β1, a marker for highly proliferative MSCs with strong chondrogenic differentiation potential, can be used to identify and isolate the desired MSC populations for therapeutic purposes [133]. In experimental studies, control, untreated OA, and OA+MSC treatment groups have been established to assess the impact of MSC-EVs on OA. By evaluating the size and quantity of MSC-EVs and examining miRNA expression profiles, these studies indicate that MSC-EVs play a role in cell therapy for OA. Additionally, MSC treatment appears to influence miRNA expression in OA, either directly or indirectly, contributing to its therapeutic effect [134,135]. These findings underline the promising potential of MSC derivatives in treating joint diseases like OA in horses.

#### 3.4.2. Application of Stem Cells in Tendon Injury in Horses

Tendon and ligament injuries are common orthopedic conditions in sports horses, significantly affecting their athletic careers and raising important animal welfare concerns [57,136]. One of the challenges in tendon injury healing is the formation of scar tissue, which impairs functional recovery and increases the risk of re-injury [49,137]. As a result, stem cell-based therapies have gained attention as a potential treatment strategy. Scientists have also evaluated the safety of allogeneic tenogenic primed mesenchymal stem cell treatments [138]. The mechanisms involved in tendon healing include the regulation of signaling pathways triggered by inflammatory cytokines, such as nuclear factor kappa B (NF-κB), which plays a crucial role in tissue repair [139,140,141].

Among the various stem cell sources, synovial mesenchymal stem cells (SM-MSCs) have shown great promise due to their superior cartilage-forming potential. Studies have demonstrated that SM-MSCs can accelerate tendon healing and improve the repair process [142,143]. However, for clinical applications, further research is required to elucidate the regulation of signaling pathways and to optimize the acquisition, culture [144], characterization, and application of mesenchymal stem cells and their secretome, including conditioned medium (CM) and extracellular vesicles (EVs) [37] in tendon injury treatment.

### 3.5. Camel Stem Cell Research

Research on camel stem cells has made significant progress, offering promising applications in regenerative medicine and biotechnology. Bactrian camel iPSCs were successfully generated for the first time using Oct4, Sox2, Klf4, and c-Myc, demonstrating pluripotency and differentiation capabilities, albeit with slower growth [145]. These cells are pivotal for future research in camel genetics and regenerative medicine. Additionally, camel MSCs, isolated from various tissues, such as bone marrow, synovial fluid, fat, muscle, and skin, exhibit multipotent differentiation into cartilage, fat, and bone cells. Notably, synovial fluid MSCs show exceptional cartilage-forming ability, surpassing bone marrow MSCs, making them particularly valuable for cartilage repair [146]. Camel skin-derived stem cells, especially spheroid progenitors, are a cost-effective resource for regenerative therapies in elite camels, aiding in wound healing, bone repair, and nerve regeneration [147]. Furthermore, camel iPSCs have key applications in cloning, germ plasm banking, and the production of humanized nanobodies for therapeutic use [148]. Embryonic stem cells (ESCs) also contribute to preserving desirable traits in elite camels, such as beauty and athleticism. These advancements highlight the potential of camel stem cell technologies in both veterinary medicine and biomedical research, particularly when combined with camelid-specific advantages like nanobody production.

**Table 1:** Summary of stem cells of herbivores and their application.

Species	Types of Stem Cells	Sources	Applications	References
Bovine	Mesenchymal Stem Cells	Adipose tissue	Adipogenic differentiation of cultivated meat	[79,80,149,150]
		Umbilical cord		[81]
		Adipose tissue	Treatment of mastitis	[75]
		Mammary gland tissue		[151]
		Bone marrow and adipose tissue		[35,56]
		Adipose tissue	Relieve follicular puncture injury	[152]
Goat	Mesenchymal Stem Cells	Adipose tissue	Treatment of mastitis	[153]
		Iliac wing	Bone regeneration	[154]
	Embryonic Stem Cells	Blastocyst	Improve infertility symptoms	[89]
Deer	Antler stem cell	Pedicle periosteum	Relieve type 1 diabetes	[55]
		Blastema tissue	Anti-aging effects	[41]
			Reducing liver fibrosis	[27]
		Reserve mesenchyme	Repair of osteochondral defects	[155]
		Blastema tissue		[120,156]
		Reserve mesenchyme	Regenerative wound healing	[7]
		Pedicle periosteum	Regeneration of alveolar bone defects	[21]
Horse	Mesenchymal Stem Cells	Synovial membrane	Treatment of tendon/ligament injuries	[143]
		Tendon		[57]
		Bone marrow	Treatment of osteoarthritis	[135]
		Muscle	Repair of laminitis	[39]
		Bone marrow	Regenerative wound healing	[157]
		Umbilical cord		[11]
		Adipose tissue	Relieve equine recurrent uveitis (ERU)	[158]
			Treatment of corneal non-healing ulcer	[159]
Camel	Mesenchymal Stem Cells	Bone marrow	Cartilage regeneration	[146]
	Induced pluripotent stem cells	Fetus	Genetic breeding of superior camels	[145]

## 4. Challenges and Limitations in Stem Cell Research

Stem cells, with their self-replicating and multi-differentiating capabilities, hold significant promise in animal research, especially in herbivores. Their applications in improving production performance, treating diseases, and enhancing animal welfare are invaluable [160]. The continuous advancement of biotechnology is expected to yield deeper insights into stem cell culture and regulatory mechanisms, leading to improved therapeutic approaches, enhanced treatment efficacy, and better safety profiles [3,161]. In addition to the applications of stem cells in tissue repair and immune modulation, scientists have also explored their applications in enhancing reproductive performance [91,162,163]. However, despite the progress, challenges, such as the technical difficulties in isolating and purifying stem cells, ensuring genetic stability during in vitro culture, the safety of induced differentiation, and addressing potential immune rejection, remain critical issues. In the future, researchers should deeply study the biological characteristics of stem cells of herbivores, explore their functions in different tissues and cell types and take donor age, sampling site, and other factors into account when obtaining stem cells [48,164,165,166]. Due to the regenerative capabilities of herbivore stem cells being greatly influenced by factors like tissue origin and in vitro culture conditions, enhancing the quantity and quality of these cells is a significant challenge for the treatment of osteoarthritis in horses. This is especially true when trying to optimize their isolation, culture, and differentiation [167]. Studying osteoarthritis in horses is challenging due to the difficulty in finding suitable experimental subjects that accurately reflect the disease’s pathology [168]. The expansion application of herbivore stem cells in regenerative medicine and disease treatment presents an optimistic challenging because the acquisition of bone marrow and fat may require invasive surgery, which will cause great pain to animals. In order to improve the transformation rate of pluripotent stem cells, further investigation into the regulatory mechanism governing in vitro reprogramming differentiation is essential to further optimize the medium composition and environmental conditions, which will be critical to advancing this field [169,170]. Herbivore stem cells have focused on short-term effects, and long-term efficacy remains uncertain [171]. Despite all these challenges, stem cell research in herbivores holds great promise. Future breakthroughs could enhance animal husbandry, improve livestock management and contribute meaningfully to biomedical science.

## 5. Conclusions

In this review, we reviewed the application of stem cells in the treatment of herbivores. Stem cell technology, with its multidirectional differentiation potential and immunomodulatory properties, provides novel therapeutic strategies for addressing a range of complex diseases in herbivores. Additionally, stem cells offer solutions to reproductive challenges, such as enhancing sperm and egg quality. In the fields of genetics and breeding, the precise modification of stem cells using gene editing technologies enables targeted cellular studies and potential therapeutic applications in herbivores. Stem cells can be used for in vitro studies of gene function and disease modeling, providing valuable insights into herbivore biology. As stem cell technology continues to evolve and interdisciplinary collaboration progresses, the future holds great promise for more widespread and in-depth applications in the field of herbivore research and industry.
